# Increased symptoms of stiffness 1 year after total knee arthroplasty are associated with a worse functional outcome and lower rate of patient satisfaction

**DOI:** 10.1007/s00167-018-4979-2

**Published:** 2018-05-10

**Authors:** N. D. Clement, M. Bardgett, D. Weir, J. Holland, D. J. Deehan

**Affiliations:** Department of Orthopaedics, Freeman Hospital, Freeman Road, High Heaton, Newcastle upon Tyne, NE7 7DD UK

**Keywords:** Total knee arthroplasty, Knee replacement, TKA, Predictors, Stiffness, Outcome

## Abstract

**Purpose:**

Symptoms of stiffness after total knee arthroplasty (TKA) cause significant morbidity, but there is limited data to facilitate identification of those most at risk after surgery. Stratifying risk can aid earlier directed treatment options.

**Methods:**

A retrospective cohort consisting of 2589 patients undergoing a primary TKA was identified from an established arthroplasty database. Patient demographics, Western Ontario and McMaster Universities Osteoarthritis Index (WOMAC), and short form (SF) 12 scores were collected pre-operatively and 1 year post-operatively. In addition, patient satisfaction was assessed for 1 year. Patients with a worse WOMAC stiffness score in 1 year were defined as the “increased” stiffness group and the other cohort as the non-stiffness group.

**Results:**

At 1 year after surgery 129 (5%) patients had a significant increase in their stiffness symptoms (20%, 95% confidence interval (CI) 17.9–22.0, *p* < 0.001), and had significantly (all *p* < 0.001) less of an improvement in their pain, function and total WOMAC scores, and SF-12 scores compared to the non-stiffness group (*n* = 2460). Patient satisfaction was significantly lower (odds ratio (OR) 0.178, CI 0.121 to 0.262, *p* < 0.001) for the increased stiffness group. Logistic regression analysis identified male gender (OR 1.66, *p* = 0.02), lung disease (OR 2.06, *p* = 0.002), diabetes (OR 1.82, *p* = 0.02), back pain (OR 1.81, *p* = 0.005), and a pre-operative stiffness score of 44 or more (OR 5.79, *p* < 0.001) were significantly predictive of increased stiffness.

**Conclusion:**

Patients with increased symptoms of stiffness after TKA have a worse functional outcome and a lower rate of patient satisfaction, and patients at risk of being in this group should be informed pre-operatively.

**Level of evidence:**

Retrospective prognostic study, Level III.

## Introduction

Total knee arthroplasty (TKA) for the treatment of end stage osteoarthritis of the knee has a patient satisfaction rate of between 80% and 90% [2]. Persistent pain and functional limitations after TKA are associated with a lower rate of patient satisfaction [12]. It is recognized that reported symptoms of stiffness, failing to squat and kneel, after surgery results in a poor outcome for the patient [17]. Approximated five percent of patients suffer stiffness as a significant complication after their TKA [6], and some require manipulation under anaesthesia [22] or revision surgery because of persistent stiffness [18]. Stiffness after knee arthroplasty may have a genetic component and epidemiological studies have found chromosomal changes in those reporting such symptoms [14], with an increased understanding of the biological basis for such a host response [16]. This is leading to a greater awareness that stiffness is potentially avoidable both from a mechanical and biological perspective [6].

The Western Ontario and McMaster Universities Osteoarthritis Index (WOMAC) [3] assesses the dimensions of pain, stiffness and function (either separately or as an overall index) [29]. The stiffness component of the WOMAC score could be used to measure patient-reported stiffness after TKA, and it has recently been demonstrated to be predictive of post-operative satisfaction [24].

The primary aim of this study was to compare the outcome (WOMAC, Short form (SF-) 12, and satisfaction) of patients with increased symptoms of stiffness 1 year after TKA with those who had no change or improvement in symptoms. The secondary aim was to identify independent predictors of increased symptoms of stiffness 1 year following TKA. The novel hypothesis is that patients with increased symptoms of stiffness have a worse outcome, and identification of independent predictors of this group would allow targeted intervention to potentially avoid increased stiffness post-operatively and improve their outcome.

## Materials and methods

**Fig. 1:**
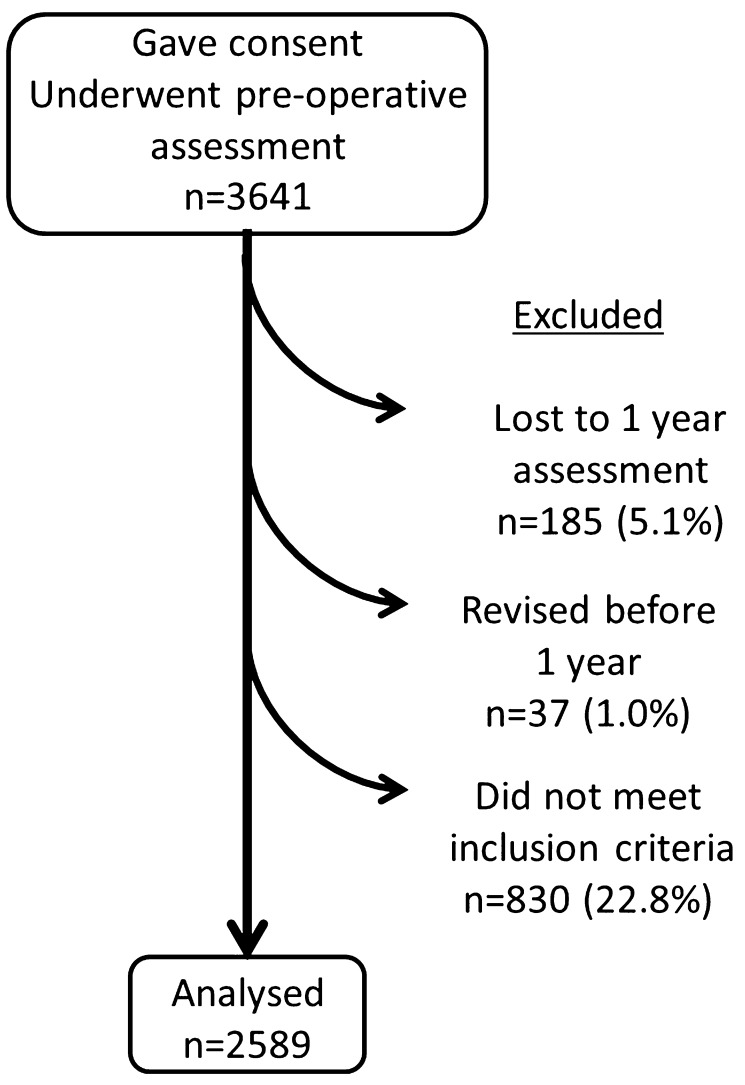
Flow diagram for the study cohort

Patients for this study were identified retrospectively from a prospectively compiled arthroplasty database held at the study centre. During a 12 year period (2003–2015) 3641 patients undergoing primary TKA at the study centre were asked to complete a pre-operative patient questionnaire. Only patients with primary osteoarthritis were included. Patients who underwent simultaneous bilateral TKA during the study period were excluded (*n* = 41) and for those patients that underwent a second TKA, after the index procedure, only the outcome of the first knee was used for analysis (*n* = 460). Patients who had a deep infection, did not complete the outcome assessments (*n* = 185), or were revised (*n* = 37) at before 1 year follow-up were also excluded from analysis. There were 2589 TKA performed during the study period with complete pre and post-operative data that met the inclusion criteria (Fig. 1). There were 1187 male patients and 1402 female patients, with a mean age of 68.9 (SD 9.7) years.

The WOMAC [3] used in this study was the Likert version 3.1 standardized with English for a British population, consisting of 24 self-administrated questions that were answered for each item on a 5-point Likert scale (none, mild, moderate, severe and extreme). It was reported as three separate subscales: pain, physical function, and stiffness. The WOMAC pain subscale had five questions scored 0 to 4 and was considered invalid if more than one item was missing; hence, it had a range of 0 (no pain) to 20 (maximal pain). In the event of a missing item, the remaining four items were averaged and then multiplied by five [5]. The WOMAC function subscale has 17 questions scored 0–4 and was considered invalid if more than three items were missing. It had a range of 0 (maximal function) to 68 (minimal function). In the event of missing items, the remaining items were averaged and then multiplied by 17. The WOMAC stiffness subscale had two items scored 0–4 and was considered invalid if either was missing; hence it had a range from 0 (no stiffness) to 8 (maximal stiffness). According to recent recommendations converted the score to a percentage where 0 is the worst and 100 is the best [19].

The Short Form (SF-) 12 is a generic assessment tool to measure a patients wellbeing, which is assessed using a physical component summary (PCS) and a mental component summary (MCS) [25]. Both the SF-12 PCS and MCS range from 0 (worst level of functioning) to 100 (best level of functioning).

Patient satisfaction was assessed by asking the question “How satisfied are you with the results of your knee replacement surgery?” at 1 year following surgery. The response was recorded using a four point Likert scale: very satisfied, somewhat satisfied, somewhat dissatisfied, and very dissatisfied. Patients who recorded very or somewhat satisfied were classified as satisfied.

Patients who had a worse or negative (1 year—pre-operative) change in the WOMAC stiffness score were defined as the increased symptoms of stiffness group. This group with increased symptoms of stiffness were compared to those who had no change or improved symptoms at 1 year. Patients that had no change or an improvement in their stiffness symptoms were used as the comparative subgroup who did not report worsened stiffness as a symptom.

The Freeman Joint Registry is an institutional audit registered with the Newcastle upon Tyne Hospitals NHS Foundation Trust since 2003 (Caldicott@nuth.nhs.uk, Audit Ref: 3290) (Patients provide written consent to participate in the audit for which patients complete patient-reported outcomes before and at multiple time points following surgery. The collection and use of audit data is approved by the Trusts Caldicott Guardian Mr A Welch (Caldicott ID:2840) at Caldicott@nuth.nhs.uk.

### Statistical analysis

Statistical analysis was performed using Statistical Package for Social Sciences version 17.0 (SPSS Inc., Chicago, IL, USA). The data assessed demonstrated a normal distribution and parametric tests were used to assess continuous variables for significant differences between groups. A Student’s *t* test, unpaired and paired were used to compare linear variables between groups. Dichotomous variables were assessed using a Chi-square test. Receiver operating characteristic (ROC) curve analysis was used to identify thresholds (cut points) in linear variables that were significantly different between the groups. The area under the ROC curve ranges from 0.5, indicating a test with no accuracy, to 1.0 where the test is perfectly accurate by identifying all satisfied patients. The threshold is equivalent to the point (WOMAC score) at which the sensitivity and specificity are maximal in predicting patient satisfaction [9]. Multivariate logistic regression analyses were used to identify independent predictors of increased symptoms of stiffness at 1 year. A *p* value of < 0.05 was defined as statistically significant.

A post hoc power calculation was performed using the WOMAC as the primary outcome. Using the defined minimal clinically important difference in the WOMAC of 15 points [8], a standard deviation (SD) of 26.5, an alpha 0.05 with 129 in the increased stiffness group and 2460 in the control group this offered a power of 100%.

## Results

One year following TKA 129 (5%) patients had an increase in their symptoms of stiffness, with a mean decrease (worse) of 20.0 [95% confidence intervals (CI) 17.9–22.0] points in the WOMAC stiffness score relative to their preoperative score.

**Fig. 2:**
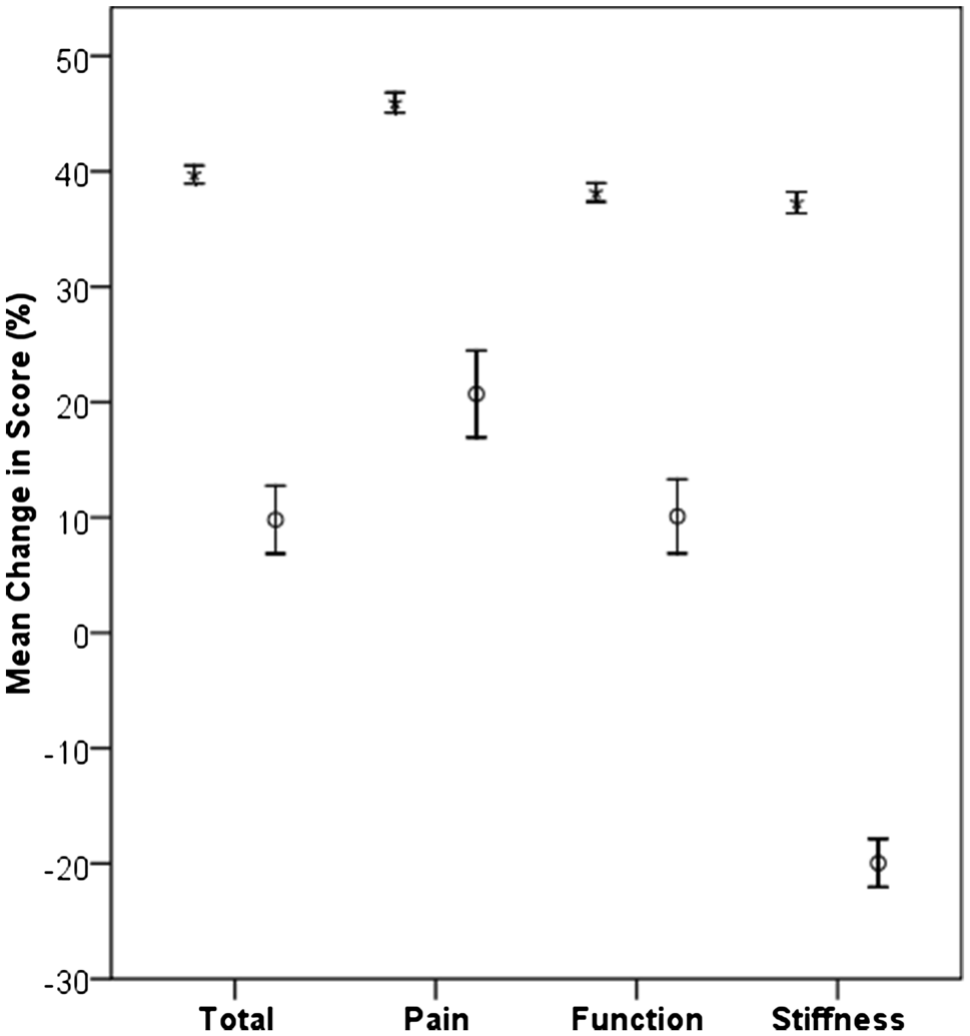
Mean change in the components and total WOMAC for those with increased stiffness (circles) and the control (star) groups. Error bars represent 95% confidence intervals

Both groups had a statistically significant improvement in the components and total WOMAC scores and the SF-12 PCS and MCS, except for the WOMAC stiffness component which deteriorated for the increased stiffness group (Table 1). Despite significant increases in all outcomes measured in the increased stiffness group, other than the stiffness WOMAC score, the non-stiffness group had a significant greater improvement. The non-stiffness group enjoyed an approximate 40% improvement in all of the components and total WOMAC scores, whereas the increased stiffness group had at best a 21% improvement in pain and at worst a 20% worsening in the stiffness component (Fig. 2). Patient satisfaction was significantly lower (odds ratio (OR) 0.178, 95% CI 0.121 to 0.262, *p* < 0.001) for the increased stiffness group (*n* = 83, 64.3%) when compared to the control group (*n* = 2229, 91.0%).

**Table 1:** Post-operative outcome measures and the difference relative to pre-operative scores for the all patients according to group

Functional Measure		Increased Stiffness		Difference	95% CI		*p* value*
		Yes (*n* = 129)	No (*n* = 2460)		Lower	Upper	
WOMAC							
Total	1 year	55.8 (22.4)	75.7 (19.5)	20.0	16.5	23.4	< 0.001
	Change (95% CI)	9.8 (6.9 to 12.7)	39.7 (38.9 to 40.5)	29.9	26.4	33.4	< 0.001
	*p* value**	< 0.001	< 0.001				
Pain	1 year	63.1 (24.0)	81.0 (19.8)	18.0	14.4	21.5	< 0.001
	Change (95% CI)	20.7 (16.9 to 24.5)	46.0 (45.1 to 46.8)	25.2	21.3	29.2	< 0.001
	*p* value**	< 0.001	< 0.001				
Function	1 year	55.4 (23.6)	74.5 (20.7)	19.1	15.4	22.8	< 0.001
	Change (95% CI)	10.1 (6.9 to 13.3)	38.2 (37.4 to 39.0)	28.1	24.2	31.7	< 0.001
	*p* value**	< 0.001	< 0.001				
Stiffness	1 year	41.0 (22.4)	73.2 (21.3)	32.3	28.5	36.0	< 0.001
	Change (95% CI)	− 20.0 (17.9 to 22.0)	37.3 (36.4 to 38.2)	57.2	53.1	61.3	< 0.001
	*p* value**	< 0.001	< 0.001				
SF-12							
PCS	1 year	31.8 (8.8)	37.9 (11.1)	6.1	4.2	8.1	< 0.001
	Change (95% CI)	3.4 (1.8 to 5.0)	10.3 (9.9 to 10.7)	6.9	5.1	8.8	< 0.001
	*p* value**	< 0.001	< 0.001				
MCS	1 year	44.0 (14.7)	49.5 (12.7)	5.5	3.2	7.8	< 0.001
	Change (95% CI)	− 2.2 (− 0.1 to − 4.4)	2.4 (1.9 to 2.9)	4.7	2.5	6.9	< 0.001
	*p* value**	0.04	< 0.001				

*t test

**Paired *t* test

**Fig. 3:**
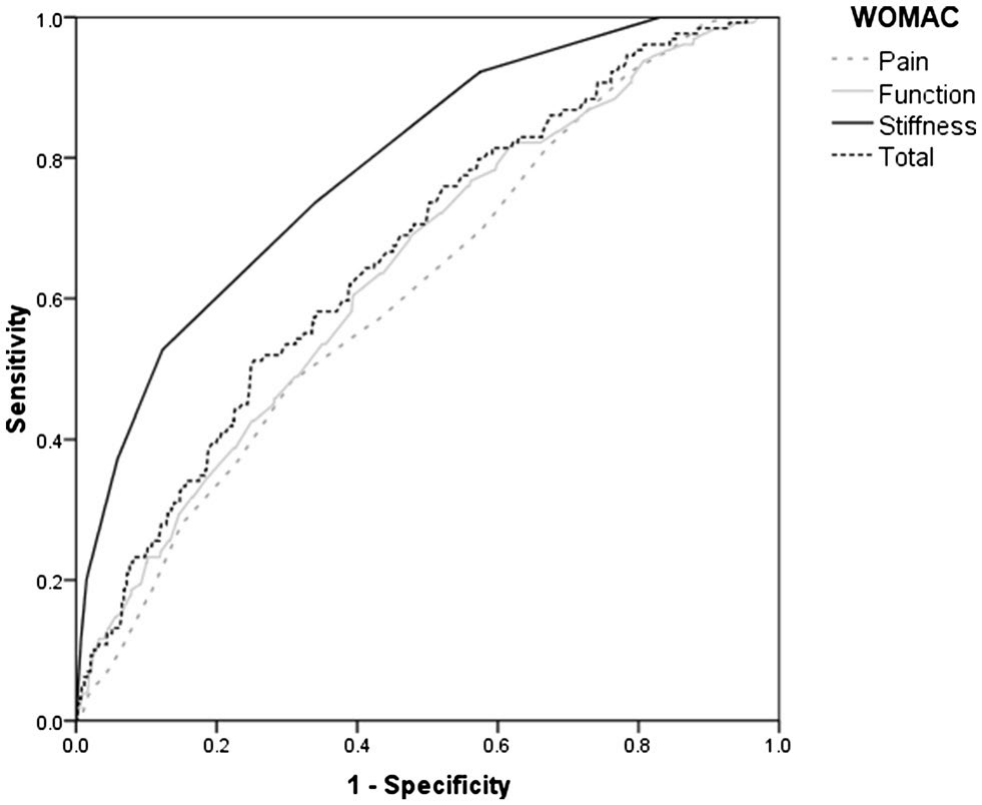
ROC curve for predicting increased stiffness 1 year after surgery using the pre-operative components and total WOMAC score

Univariate analysis identified pre-operative factors that were predictive of increased symptoms of stiffness at 1 year (Table 2). ROC curve analysis was used to identify threshold values in the linear variables that were demonstrated to be significantly different between the groups (Table 2). The most reliable predictor of increased stiffness at 1 year was the pre-operative WOMAC stiffness score (Fig. 3) (Table 3). Interestingly pain, function, and the total WOMAC scores were poor predictors, with an AUC of less than 0.7. The threshold values were used as dichotomous variables as predictors in the regression models. Logistic regression analysis identified male gender (*p* = 0.017), lung disease (*p* = 0.002), diabetes (*p* = 0.02), back pain (*p* = 0.005), and a pre-operative stiffness score of 44 or more (*p* < 0.001) were significantly predictive of increased stiffness 1 year following surgery (Table 4).

**Table 2:** Patient demographics and pre-operative functional scores according to symptoms of stiffness 1 year after surgery

Demographic	Descriptive	Increased Stiffness		Odds ratio/*difference*	95% CI		*p* value
		Yes (*n* = 129)	No (*n* = 2460)		Lower	Upper	
Gender (*n*, % of group)	Male	78 (60.4)	1109 (45.1)	1.86	1.30	2.68	0.001
	Female	51 (39.5)	1351 (54.9)				
Mean age (years: mean, SD)		69.7 (9.6)	68.8 (9.7)	*0.9*	− 2.6	8.7	n.s
BMI (kg/m^2^: mean, SD)		29.1 (5.0)	29.8 (6.9)	*0.7*	− 0.6	1.9	n.s
Comorbidity (*n*, % of group)	Heart disease	21 (16.3)	414 (16.8)	0.96	0.60	1.55	n.s
	Hypertension	66 (51.2)	1346 (54.7)	0.87	0.61	1.24	n.s
	Lung disease	33 (25.6)	366 (14.9)	1.97	1.30	2.97	0.001
	Cancer	8 (6.2)	117 (4.8)	1.32	0.63	2.77	n.s
	Neurological disease	14 (10.9)	139 (5.7)	2.03	1.14	3.63	0.02
	Diabetes mellitus	27 (20.9)	338 (13.7)	1.66	1.07	2.58	0.02
	Gastric ulceration	18 (14.0)	309 (12.6)	1.13	0.68	1.88	n.s
	Kidney disease	8 (6.2)	71 (2.9)	2.23	1.05	4.73	0.03
	Liver disease	5 (3.9)	39 (1.6)	2.50	1.0	6.46	0.05
	Anaemia	9 (7.0)	237 (9.6)	0.70	0.35	1.40	n.s
	Depression	28 (21.7)	348 (14.1)	1.68	1.09	2.60	0.02
	Back pain	76 (58.9)	1233 (50.1)	1.43	1.0	2.0	n.s
Functional measures (mean, SD)							
WOMAC	Total	46.0 (16.1)	36.0 (16.2)	*10.0*	7.1	12.8	< 0.001
	Pain	42.4 (15.9)	35.1 (17.7)	*7.3*	4.2	10.4	< 0.001
	Function	45.3 (16.9)	36.3 (16.9)	*9.0*	6.0	12.0	< 0.001
	Stiffness	61.0 (22.4)	36.0 (19.6)	*25.0*	21.5	28.5	< 0.001
**SF-12**	PCS	28.4 (7.6)	27.5 (7.4)	*0.8*	− 0.5	2.2	n.s
	MCS	46.3 (13.3)	47.1 (13.6)	*0.8*	−1.6	3.2	n.s

n.s. non-significant

*Unpaired *t* test unless otherwise stated

**Chi-square

**Table 3:** ROC curve analysis identifying the threshold value for the components and the total WOMAC scores that predict increased stiffness at 1 year

WOMAC	Threshold value	Sensitivity	Specificity	AUC	95% CI		*p* value
					Lower	Lower	
Total	40	61.2	61.2	0.666	0.620	0.712	< 0.001
Pain	36	56.6	57.8	0.616	0.569	0.663	< 0.001
Function	40	60.5	60.5	0.643	0.596	0.690	< 0.001
Stiffness	44	73.6	66.0	0.790	0.751	0.829	< 0.001

**Table 4:** Patient demographics that are independent predictors of increased symptoms of stiffness after TKR using bivariate regression analysis

Demographic	Descriptive		OR	95% CI		*p* value
				Lower	Upper	
Gender	Male		Reference			
	Female		0.604	0.398	0.915	0.017
Mean age			1.0	0.978	1.022	n.s
BMI			0.983	0.941	1.026	n.s
Comorbidity	Not present		Reference			
	Heart disease		0.669	0.390	1.147	n.s
	Hypertension		0.788	0.524	1.184	n.s
	Lung disease		2.064	1.299	3.279	0.002
	Cancer		1.086	0.478	2.469	n.s
	Neurological disease		1.684	0.852	3.329	n.s
	Diabetes mellitus		1.815	1.099	2.998	0.02
	Gastric ulceration		0.972	0.54	1.748	n.s
	Kidney disease		1.451	0.576	3.655	n.s
	Liver disease		2.341	0.765	7.162	n.s
	Anaemia		0.602	0.278	1.306	n.s
	Depression		1.499	0.863	2.603	n.s
	Back pain		1.806	1.195	2.729	0.005
Functional measure						
WOMAC	Total	< 40	Reference			
		≥ 40	0.816	0.3	2.222	n.s
	Pain	< 36	Reference			
		≥ 36	0.849	0.478	1.506	n.s
	Function	< 40	Reference			
		≥ 40	1.942	0.81	4.654	n.s
	Stiffness	< 44	Reference			
		≥ 44	5.787	3.479	9.624	< 0.001
SF-12	PCS		0.974	0.945	1.003	n.s
	MCS		0.987	0.97	1.003	n.s

All variables from Table 1 and threshold values for the components and total WOMAC score (Table 3) were all entered into the model using “enter” methodology (Nagelkerke *R*^2^ = 0.17)

*n.s*. non-significant

## Discussion

The important findings of the study were that patients with subjectively increase symptoms of stiffness have a worse functional outcome and a lower rate of post-operative satisfaction, and that male gender, lung disease, diabetes, back pain, and a pre-operative WOMAC stiffness score of 44 or more were predictive of this group.

Arthrofibrosis is a significant complication following TKA, which is reported to have an incidence of between 1 and 13% [6]. Post-operative fibrosis of the knee is defined as a limited range of movement (in flexion and/or extension), that is not attributable to specific cause, but due to soft-tissue fibrosis that was not present pre-operatively [13]. The current study assessed symptoms of stiffness as defined by the patient using the WOMAC score and this may not relate to a limited range of movement and a secondary cause was not ruled out. Loss of motion may be predictable after knee arthroplasty, but such objective findings may not necessarily relate to symptoms of stiffness [15]. Stiffness may correlate with other symptoms, but not necessarily key components of function and as such could be regarded as a distinct entity within the complex of reported measures of outcome [26, 27].

Fulfilment of patient expectations after TKA is associated with a greater rate of satisfaction [17]. Approximately 60% of patients expect to kneel and 50% expect to squat after their TKA surgery, but the likelihood of these being fulfilled at 1 year is approximately 15% and 25%, respectively [7]. When these expectations are not achieved the likelihood of the patient being dissatisfied is significantly increased (OR of 8 and 9, respectively) [7]. The risk factors in the current study could be used to identify patients at risk of increased stiffness who may then benefit from expectation modification that may improve their satisfaction.

It is interesting that lung disease, diabetes, and back pain were independent predictors of increased stiffness after TKA, as all have been associated with fibrotic or inflammatory pathologies previously. Chronic lung disease is associated with pulmonary fibrosis and this may explain why such patients are at risk of increased stiffness after TKA; the association has previously suggested [1]. Diabetes is a recognised comorbidity associated with a worse functional outcome [4] and stiffness [10] after TKA, which supports the findings of the current study. Nonspecific lower lumbar back pain has recently been demonstrated to be directly related to increased lumber stiffness [28], and although there is no link with a fibrotic condition there is an accepted inflammatory element which may be associated with the aetiology of knee stiffness [23].

A pre-operative WOMAC stiffness score of 44 or more was a significant predictor of increased symptoms of stiffness 1 year following TKA, and was demonstrated to be reliable with a AUC of 0.8. This score could be used as a screening tool, being composed of only two questions it would be simple to assess. This combined with the other risk factors identified could be used to identify an “at risk group” pre-operatively, who may benefit from peri-operative interventions or at least made aware during the consent process that they are at risk of increased symptoms of stiffness and are less likely to be satisfied. There are multiple post-operative interventions suggested to prevent stiffness from occurring [6]. However, with greater understanding of fibrosis pathways there may be inhibitors that may stop the post-operative stiffness from developing [1], which may improve the patients functional outcome and satisfaction.

The major limitation of this study was the retrospective design that did not enable range of movement data to be assessed. Recording the range of movement pre- and post-operatively would have been desirable as this could have been correlated with the patient’s assessment of their stiffness. Surgeons often define knee stiffness as limitation in the range of motion of the joint [13], but patients may not necessarily define this in the same way [11]. However, it has previously been demonstrated that a significant correlation exists between the flexion, but not extension, and the stiffness component of the WOMAC score [21]. A prospective study would have allowed subgroup assessment of patients with increased symptoms of stiffness to a greater depth than the patient-reported outcome measures used in the current study. Objective and qualitative assessment would have enable the stiffness factor to be assessed using range of movement to confirm the patients impressions or whether this was an expectation mismatch, respectively. Qualitative assessment would have also given insight into why those patients with increased symptoms of stiffness were dissatisfied with their TKA.

Surgeons should be aware that a proportion of patients will have increased symptoms of stiffness after TKA and that they have a worse functional outcome and a lower rate of post-operative satisfaction. Patients at risk may benefit from increased early physiotherapy or other treatment modalities [20] to prevent an increase in their symptoms of stiffness after TKA.

## Conclusion

Patients with increased symptoms of stiffness after TKA have a worse functional outcome and a lower rate of patient satisfaction, and patients at risk of being in this group should be informed preoperatively.
